# Airborne transmission efficiency of SARS-CoV-2 in Syrian hamsters is not influenced by environmental conditions

**DOI:** 10.1038/s44298-023-00011-3

**Published:** 2024-01-09

**Authors:** Claude Kwe Yinda, Julia R. Port, Trenton Bushmaker, Jonathan E. Schulz, Shane Gallogly, Robert J. Fischer, Vincent J. Munster

**Affiliations:** Laboratory of Virology, Division of Intramural Research, National Institute of Allergy and Infectious Diseases, National Institutes of Health, Hamilton, MT USA

**Keywords:** Microbiology, Virology, SARS-CoV-2, Viral transmission

## Abstract

Several human respiratory viruses display a clear seasonal pattern with a higher incidence in the winter season in temperate regions. We previously determined that SARS-CoV-2 is more stable at low-temperature and low-humidity conditions compared to warmer temperature and higher-humidity. To determine if this translates into differential airborne transmission rates in vivo, we performed airborne transmission experiments in the Syrian hamster model under three different environmental conditions (10 °C, 45% relative humidity (RH), 22 °C, 45% RH, and 27 °C, 65% RH). We compared the ancestral SARS-CoV-2 Lineage A with the more transmissible Delta Variant of Concern (VOC). Airborne transmission was evaluated using SARS-CoV-2 infected donor animals at 24 h post inoculation. Sentinels were placed at a 90 cm distance in a transmission set-up and exposed for 1-h to infected donor animals. While environmental conditions moderately impacted lung RNA titers, the shedding kinetics of the donors were not affected by the environmental conditions and did not differ significantly between variants on day 1. Overall, the highest transmission efficiency was observed at 22 °C, 40%RH for Delta (62.5%, based on seroconversion), and ranged between 37.5 and 50% for all other conditions. However, these differences were not significant. To elucidate this further, we performed aerosol stability comparisons and found that infectious virus remained stable during a 1-h time window across all conditions. Our data suggest that even when environmental conditions affect the stability of SARS-CoV-2, this may not directly be translatable to measurable impacts on transmission in an experimental setting when exposure time is restricted.

## Introduction

Environmental stability of SARS-CoV-2 in aerosols and on surfaces is affected by variables such as temperature and relative humidity^1,2^. SARS-CoV-2 is more stable at low temperature and low humidity than at higher temperature and low humidity on surfaces and in aerosols^3–5^. Epidemiological modeling studies have suggested that changes in temperature and humidity may affect SARS-CoV-2 transmission^6,7^. Human coronaviruses (HCoVs); HCoV-NL63, HCoV-229E, HCoV-HKU1, andHCoV-OC43 are endemic in the human population and display seasonal patterns, generally peaking during December–March in the USA^8^. More broadly, HCoVs display the highest prevalence during the winter in temperate regions^9–11^, whereas in tropical regions HCoV seasonality is less predictable^12,13^. The seasonality of HCoVs is likely the result of a combination of viral, host, and environmental factors. Lower temperatures improve the stability of coronaviruses^2^, increasing the likelihood of the host coming in touch with the infectious virus. In addition, colder temperatures can result in behavior change, including an increase in indoor human contact during winter^14,15^.

Transmission of SARS-CoV-2 in human population is influenced by environmental factors through four major interlinking mechanisms: increased risk of preexisting conditions associated with disease severity; immune system impairment; viral environmental survival; and behaviors that increase viral exposure^16–18^. Epidemiologic evidence suggests that SARS-CoV-2 transmission risk is higher at lower ambient temperatures and humidity, indicating a potential seasonality of SARS-CoV-2 transmission^6,7,19–25^. However, these studies and correlations were performed in the presence of unmeasured confounders. Here, we investigated the direct impact of environmental conditions on the aerosol transmission efficiency of SARS-CoV-2 in the well-established Syrian golden hamster model. The Syrian hamster model has been extensively used by different groups to understand routes of transmission and increased transmission potential of VOCs^26–31^.

## Results

### Minimal impact of environmental conditions on hamsters

**Fig. 1:**
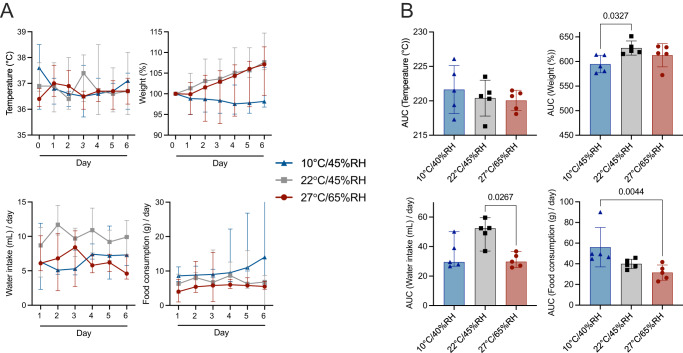
Environmental impact assessment of the conditions on temperature, weight, water, and food intake of hamsters. To acclimatize the animals to study conditions, animals were housed in airborne transmission cages^30^ inside an environmental chamber. Hamsters were first acclimatized by gradual in- or decrease of relative humidity and temperature and subsequently housed at either 10 °C, 45% RH, 27 °C, 65% RH or 22 °C, 45% RH for 5 days before the start of the experiment. **A** Changes in hamster activity and physiology during acclimatization to environmental test conditions. Body temperature, weights, food uptake and water uptake were measured daily. Median and 95%CI are depicted in line graphs, *N* = 5. blue = 10 °C, 45% RH, red = 27 °C, 65% RH, gray = 22 °C, 45% RH. **B** Area under the curve (AUC) for body temperature, weights, food uptake and water uptake. Median and 95%CI are depicted in bar graphs, *n* = 5. blue = 10 °C, 45% RH, red = 27 °C, 65% RH, gray = 22 °C, 45% RH. *P* values are indicated where significant. Abbreviations: AUC area under the curve.

We focused on three environmental conditions that recapitulate the extremes of seasonal temperatures in the Syrian hamster model^32^. We chose climate controlled indoor conditions (22 °C, 45%RH) as the control, and compared this to temperate fall (10 °C, 45 %RH) and tropical condition (27 °C, 65%RH) (Fig. 1A).

First, we investigated the effect of environmental changes on hamster behavior and activity (Fig. 1A, B). Hamsters at 27 °C, 65%RH showed slightly less physical activity and huddling compared to the other two groups. No overt differences in activity, mental awareness, nor signs of distress were observed. We measured body temperature, weight, food, and water intake over a period of 6 days. Median body temperature for animals at temperate, climate controlled indoor, and tropical conditions was 36.8 °C, 36.7 °C, and 36.9 °C, respectively (range: 35.8–38.5 °C, 35.7–38.5 °C, respectively, *n* = 5). Cumulative body temperatures measured by calculating area under the curve (AUC) showed no significant differences at different environmental conditions (*p* > 0.999, *n* = 5, Kruskal-Wallis test followed by Dunn’s multiple comparisons test) (Fig. 1B). Animals at temperate fall conditions maintained their weight below baseline median of 98.8% (range: 97.5–98.8, *n* = 5), while animals at indoor and tropical conditions continuously gained weight, median 103.9% and 102.7%, respectively (range: 99.6–114.7% and 92.8–111.4%, *n* = 5), while Cumulative weight gain/loss was only significantly different between animals at normal and temperate fall conditions (*p* = 0.0327, *n* = 5, Kruskal-Wallis test followed by Dunn’s multiple comparisons test) (Fig. 1B). Environmental temperature and humidity affected water and food consumption. The median amount of water consumed was highest for animals at climate controlled indoor condition (median = 10.0 mL/day, range 4.1–14.5 mL/day, *n* = 5) followed by animals in the temperate fall group (median = 7.1 mL/day, range 2.3–11.9 mL/day, *n* = 5) and those tropical group (median = 6.2 mL/day, range = 2.1–10.8 mL/day, *n* = 5). Overall, animals at climate controlled indoor conditions significantly consumed more water than those at tropical conditions (*p* = 0.0267, *n* = 5, Kruskal-Wallis test followed by Dunn’s multiple comparisons test) (Fig. 1B). Animals at temperate fall condition consumed the highest amount of food daily, (median = 11.3 g/day, range 6–32 g/day, *n* = 5), followed by animals at climate controlled indoor and tropical conditions with a median food consumption of 7.6 and 6.09 g/day (range = −2–16.1 and 1–15.4 g/day). However, the cumulative food intake was only statistically significant between animals at tropical and climate-controlled indoor conditions (*p* = 0.0044, *n* = 5, Kruskal-Wallis test followed by Dunn’s multiple comparisons test). Taken together, extreme environmental conditions did not cause overt changes in hamsters’ behavior and activity.

### Hamster transmission cage set-up allows largely fine particles from donor to sentinel cage

**Fig. 2:**
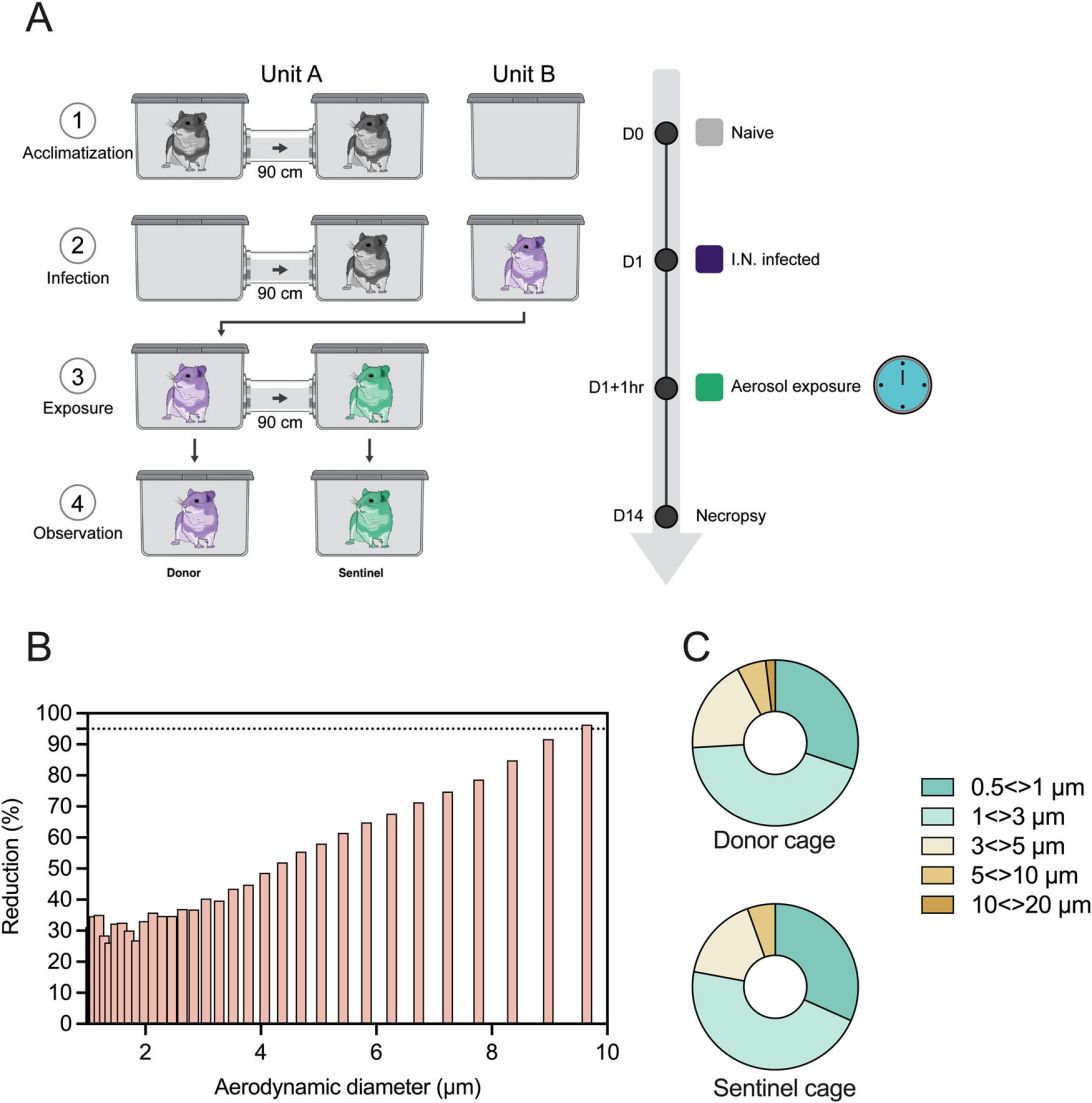
Study scheme and particle size distribution in the transmission cage set-up. **A**. Schematic visualization of the transmission cage set-up at 90 cm. Transmission cages were designed to model airborne transmission between Syrian hamsters at 90 cm distance. Droplets were generated by spraying a 20% glycerol/water solution into the donor cage. The size of particles traveling between donor and sentinel cages and particle reduction by aerodynamic diameter between donor and sentinel cages was determined (**B**). The dotted line represents 95% reduction in particles. The aerodynamic diameter was 1–10 μm. Particle distribution detected in each donor and sentinel cage was also determined (**C**).

The caging system used in this study is described in ref. ^30^. The spacing between the donor and sentinel cages was kept at 90 cm. We employed an aerodynamic particle sizer to quantify the aerodynamic size of particles (dynamic range of 0.5–20 µm) traveling from donor to sentinel cage at this distance (Fig. 2A). Droplets and aerosols were generated in the donor cage (20% (v/v) glycerol solution, sprayed with a standard spray bottle and the particle size profile was determined at the beginning and end of the connecting tube to study the potential for size exclusion when an airflow of 30 cage changes/h was applied. At this distance, 75% of particles ≥5 µm did not traverse into the sentinel cage and almost no particle ≥10 µm were detected (99.9% reduction). Hence, while in the donor cage 2.0% of detected particles were 10 µm, in comparison the particle profile in the sentinel cage contained <0.01% particles >10 µm (Fig. 2B, C). The overall absence of particles ≥10 µm and extensive reduction of particles 5–10 µm indicate that the caging system at this distance is suitable to study airborne transmission that occurs primarily via fine aerosols.

### Minimal impact of SARS-CoV-2 shedding under different environmental conditions

**Fig. 3:**
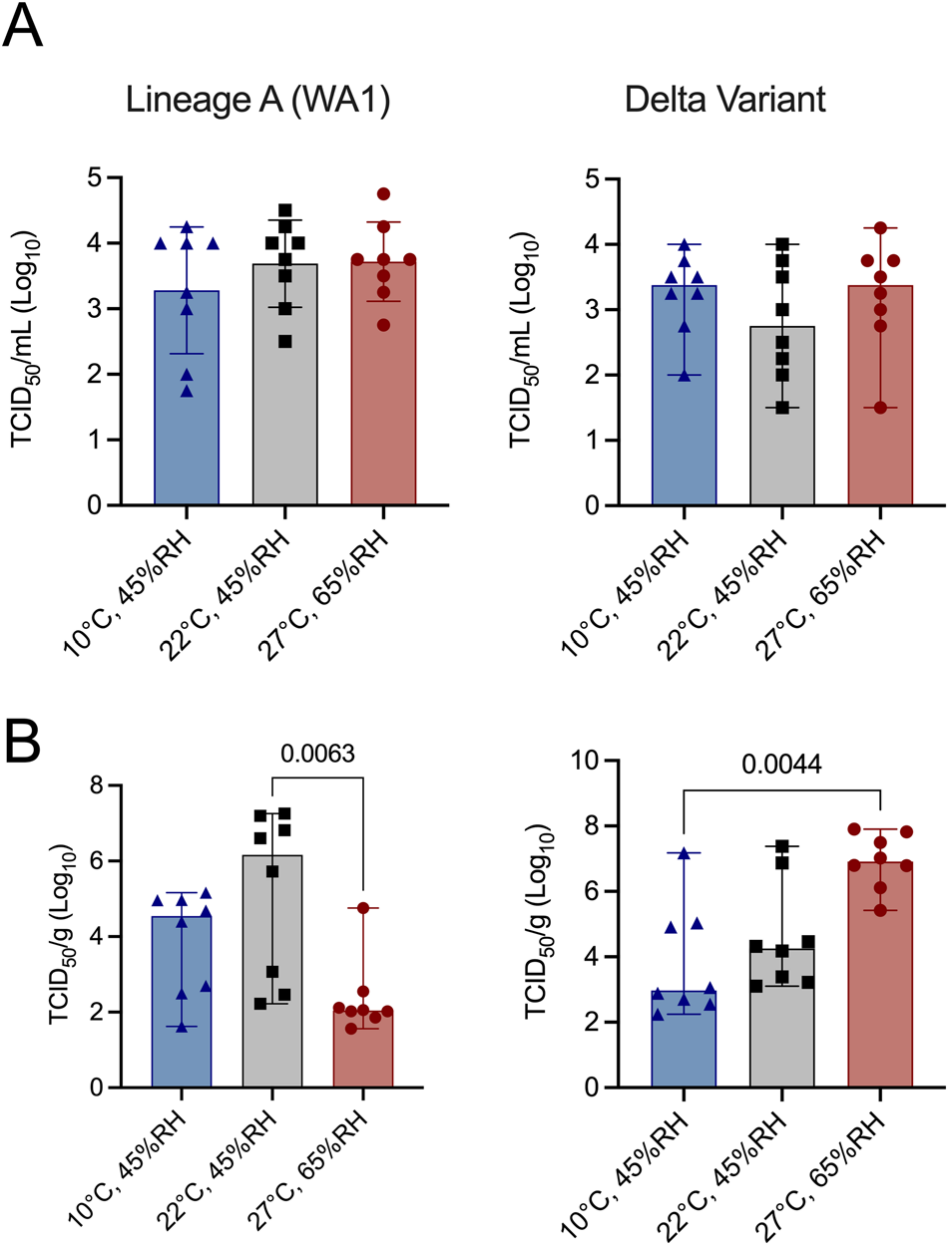
SARS-CoV-2 shedding and lung viral load. Donor Syrian hamsters were inoculated with either SARS-CoV-2 Lineage A or Delta at 8 × 10^4^ TCID_50_ via the intranasal route. Upper and lower respiratory tract SARS-CoV-2 replication in donor animals on day 1 post inoculation. **A** Infectious virus titer in oropharyngeal swabs. **B** Infectious virus titer in lungs. Bar graphs depicting median, 95% CI, and individual values, *n* = 8, ordinary two-way ANOVA, followed by Šídák’s multiple comparisons test. blue = 10 °C, 45% RH, red = 27 °C, 65% RH, gray = 22 °C, 45% RH. *P* values are indicated where significant. Abbreviations: RH relative humidity, TCID tissue culture infectious dose.

Donor animals were acclimatized over a period of five days at their respective environmental conditions. Donor animals were inoculated intranasally (IN) with 8 × 10^4^ TCID_50_, of either Lineage A or the Delta variant of concern. To assess the impact of the environmental condition on the shedding, we measured infectious virus titers in the upper respiratory tract (oropharyngeal swabs) and in the lower respiratory tract (lungs) after 1 day post inoculation (DPI). The donors infected with Lineage A shed on average 3.3 TCID_50_/mL (Log_10_) (temperate condition, *n* = 8) or 3.7 TCID_50_/mL (Log_10_) (controlled indoor conditions and tropical climate, *n* = 8) while oropharyngeal swabs of hamsters inoculated with the Delta variant had 3.4, 2.8, and 3.4 TCID_50_/mL (Log_10_) for temperate, climate controlled indoor, and tropical conditions, respectively (*n* = 8) (Fig. 3A). Virus titers in the lower respiratory tract of the donors were more variable (Fig. 3B). For Lineage A inoculated animals, donors at climate controlled indoor conditions had the highest amount of virus titers in their lungs followed by temperate and tropical conditions (median = 5.2, 3.9 and 2.4 TCID_50_/g (Log_10_), respectively). Nevertheless, a statistically significant difference was only observed between animals under tropical and normal environmental conditions (*p* = 0.0063, *n* = 8, Kruskal-Wallis test followed by Dunn’s multiple comparisons test). This was not observed for hamsters inoculated with the Delta variant. In this group, animals at tropical conditions had higher virus titers, followed by hamsters at climate-controlled indoor condition, and then temperate condition (6.9, 4.6, and 3.8 TCID_50_/g (Log_10_), respectively). Statistically significant differences in lung viral titers were observed between tropical and temperate (*p* = 0.0044, *n* = 8, Kruskal-Wallis test followed by Dunn’s multiple comparisons test).

### Minimal impact of environmental conditions on SARS-CoV-2 transmission

**Fig. 4:**
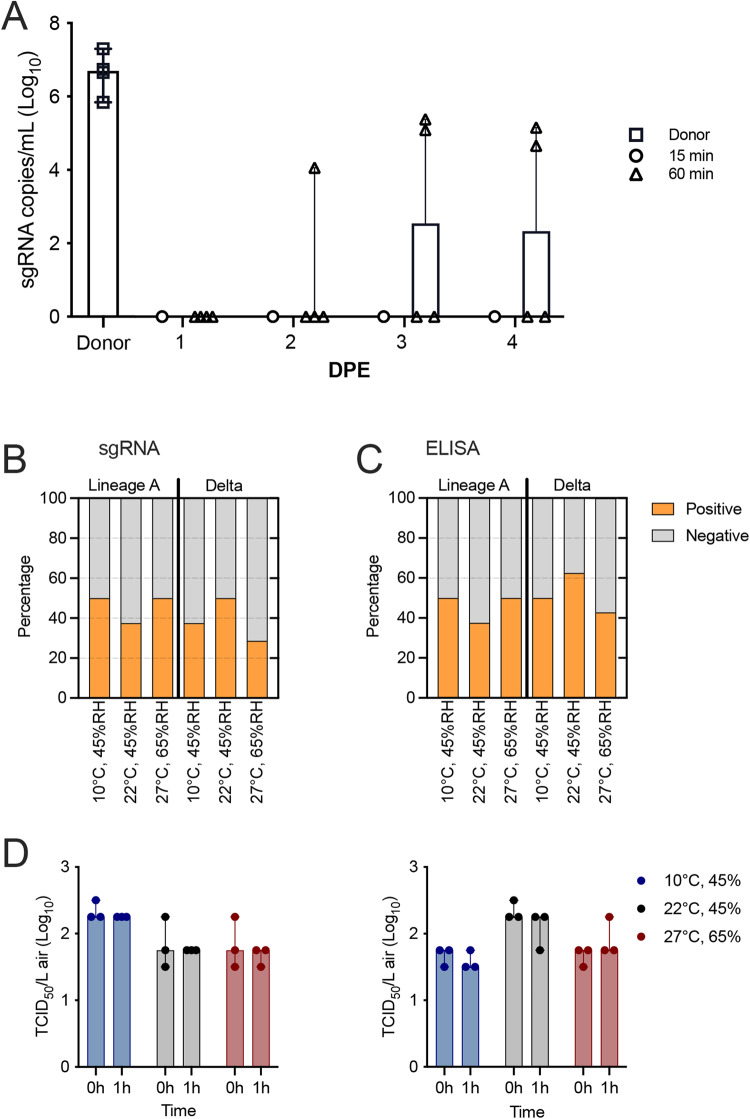
Efficiency of airborne transmission of Lineage A and Delta at different environmental conditions. Donor Syrian hamsters were inoculated with either SARS-CoV-2 Lineage A or Delta at 8×10^4^ TCID_50_ via the intranasal route. Sentinel animals were exposed through the air at 1 day post inoculation under different environmental conditions at a 1:1 ratio. **A** Exposure at 90 cm distance, at climate controlled indoor conditions for 15 min (white) and 1 h (black). Infection success and transmission efficiency was measured by upper respiratory tract shedding of sgRNA in oral swabs from individual donors at 1 day post infection and sentinels 1–4 days post exposure, respectively. Bar graph depicts median, 95% CI, and individuals, *n* = 4. Summary of transmission efficiency at 90 cm distance and during a 1-h exposure window measured by sgRNA in oropharyngeal swabs (**B**) and seroconversion assessed by anti-spike ELISA (**C**). **D** Environmental stability of aerosolized SARS-CoV-2 Lineage A and Delta variants. Data represent three independent replicates performed for each of the environmental condition in a 1 h run. The titer of aerosolized viable virus is expressed in 50% tissue-culture infectious dose per liter of air. Dots depicting values from independent runs and bars represent median with 95% CI, *n* = 3, Wilcoxon matched-pairs signed rank test. blue = 10 °C, 45% RH, gray = 22 °C, 45% RH, red = 27 °C, 65% RH. Abbreviations: RH relative humidity, TCID tissue culture infectious dose, sg subgenomic, DPE day post exposure.

Effects of environmental conditions are difficult to detect when overall transmission efficiencies are saturated. Therefore, we first determined the time window under which the airborne transmission is not saturated (25-70% transmission). First, donor animals were inoculated IN with 8 × 10^4^ TCID_50_ of Lineage A SARS-CoV-2 and in a 1:1 ratio, at 1 DPI, donor animals were exposed to two sets of naive sentinels: the first set for 15 min, and the second set for an hour. The transmission set-up was maintained at 90 cm distance between infected and naïve animals. The sentinels were swabbed daily for four days post exposure (DPE) and sgRNA was quantified. With 15 min exposure, no animal was sgRNA positive up till 4 DPE, while with 1 h exposure, one out of four animals was sgRNA positive at 2 DPE. At 3 and 4 DPE, two sentinels were sgRNA positive (Fig. 4A). We concluded that a 1-h exposure of sentinels represented a condition at which transmission efficiencies are not saturated and, hence, useful to investigate the effect of environmental changes on transmission.

With the outcome above, we then examined the transmission of Lineage A compared with the Delta variant at the three different environmental conditions. Four out of eight (4/8), 3/8, and 4/8 sentinel hamsters shed sgRNA by two DPE for temperate fall, climate-controlled indoor, and tropical conditions, respectively. When exposed to donors infected with the Delta variant, 3/8, 4/8, and 2/7 sentinel hamsters shed sgRNA by 2 DPE for temperate fall, controlled laboratory, and tropical conditions, respectively. (Fig. 4B, Supplementary Table 1).

We confirmed the transmission efficiency by investigating seroconversion (Fig. 4C, Supplementary Table 1). Interestingly, while all animals with anti-SARS-CoV-2 spike antibodies had sgRNA-positive swabs for all Lineage A exposed sentinels, in the Delta group, we found that 4/8, 5/8, and 3/7 animals had seroconverted for temperate fall, controlled laboratory and tropical conditions, respectively. Overall, hamsters exposed to the Delta variant at controlled lab condition had the highest rate of transmission (62.5%, based on seroconversion) compared to other conditions (40–50%).

### 1-h aerosol stability of SARS-CoV-2 not impacted by environmental condition

To examine why changes in environmental conditions did not significantly influence the airborne transmission efficiency, we investigated the stability of aerosolized SARS-CoV-2 Delta and Lineage A variants at the different environmental condition over the duration of 1 h^33^. Overall, limited impact of the environmental conditions was observed between the starting infectious dose and the infectious dose after 1 h in suspension in the Goldberg drum. Slight variation in starting dose were observed between conditions. The average infectious titers for Lineage A at time zero were 2.3, 1.8, and 1.8 TCID_50_/L of air (Log_10_) for temperate, climate controlled indoor and tropical conditions, respectively, and at 1 h the amount of viable virus either remain the same or changed slightly to 2.3, 1.8, and 1.7 TCID_50_/L of air, respectively (Fig. 4D). We observed the same trend with the Delta variant: the average infectious virus at the beginning was 1.8 TCID_50_/L of air (Log10) for all conditions, and at 1 h, the amount of infectious virus either remain the same or changed slightly to 1.7, 1.8, and 1.7 TCID_50_/L of air, respectively. This indicated that no significant change in aerosolized SARS-CoV-2 stability was observed within 1 h of exposure.

## Discussion

Respiratory viruses display a seasonal pattern, with a higher incidence during fall and winter. Influenza A virus, for example, has significant increased during the winter season, whereas low activity is detected during the summer months^34^. Human coronaviruses (OC43, 229E, NL63, and HKU1), on the other hand, circulate only in the winter and spring in areas with temperate climate, and throughout the year in tropical regions^13,35,36^. In the case of SARS-CoV-2, epidemiological data suggests that environmental factors influence the transmission kinetics of the virus^6,24,25^. However, these data are reported in the presence of other confounding factors such as changing mitigation strategies and host immunity.

In this study, we used the Syrian hamster model to investigate the effect of environmental conditions on the aerosol transmission of SARS-CoV-2. Our data showed that the transmission was more efficient with Delta variant at 22 °C, 45%RH, consistent with epidemiological data that showed this variant gained high transmissibility and was able to outcompete previous variants^37^. This transmission advantage was based on seroconversion status at day 14 and not with oral swab sub-genomic (sg)RNA loads collected on 1–3 DPE. This means that in hamsters, transmission experiments require serology as the final benchmark to assess transmission efficiency^38^. The increase in transmissibility over 90 cm distance in the hamster between Delta and Lineage A was only observed after seroconversion, which supports epidemiological studies in humans^39^, as well as transmission studies in hamsters^26^. However, this experimental set-up was not directly designed to study differences in intrinsic transmission potential between viruses. For this, an experimental design which allows for viruses to compete against each other in transmission chains, therefore mimicking population-based transmission more closely would be needed. Increased transmissibility can be a result of increased shedding, which has been shown for Delta over Lineage A in humans^40^. Here, the focus was on the effect of the environmental condition during a short 1 h transmission event, not the susceptibility to infection as such.

Furthermore, it has been established for influenza A viruses that aerosol transmission is a consequence primarily of replication in the upper respiratory tract^41^. While the absence of significant differences in donor shedding as measured by oropharyngeal swabs and in the subsequent transmission in this model suggest that aerosol transmission is predominantly caused by upper respiratory tract replication, this cannot be concluded from this data. Oropharyngeal swab positivity is not always evidence for infectious virus exhalation in the air^42^, and mostly likely such swabs capture virus from all respiratory compartments, and not the nasal turbinates only. More in depth within-host kinetics need to be established to determine from where exhaled virus originates.

It has been proposed that exposing animals to temperatures below normal can result in increased energy demand to generate heat, which may have a negative effect on the immune system^9^, resulting in alterations in infection susceptibility. Contrarily, we observed only subtle differences in transmission efficiency at different environmental conditions. Virus shedding from donor hamsters at 1 DPI did not differ significantly under the investigated environmental conditions. In addition, we found that during a 1-h exposure window, there are only subtle differences in the stability of the virus in the aerosol. Contrarily, Chan et al.^43^ observed that low environmental temperatures increased the degree of virus shedding and exacerbated the disease, suggesting that higher transmission potential is expected for hamsters at low temperatures. However, this was mostly observed at 7-day DPI. Ganti and colleagues observed that when exposures were carried out with optimal timing and a high inoculation dose, relative humidity and temperature had no effect on transmission, whereas at sub-optimal exposure timing and a lower inoculation dose, they noticed improved SARS-CoV-2 transmission at high relative humidity or high temperature^23^. Their findings may be explained in part by the virus’s apparent stability in high relative humidity^1^. Moreover, their rodent transmission set-up was made of cages modified through the addition of a double-walled porous barrier. This suggests that the air passing through the porous barrier includes a high concentration of large particles, which react differently to changes in relative humidity than small particles^1,44^. In addition, our set-up allowed only for aerosol transmission with a donor-sentinel sub-optimal exposure of 1 h, which is more practical given that in human population infection have been observed to occur within the first hour of exposure^45–47^. The finding of all these studies suggest that different experimental set-ups can influence the outcome of transmission efficiency at different environmental conditions.

One limitation of our study was the low animal numbers. Even though more animals would have provided a clearer picture of the effect of temperature and RH on transmission, the logistics of acclimating and re-acclimating animals in customized transmission cages in an environmental chamber limit the number of animals that can be handled at any given time. Another way to circumvent this would have been to make use of an airborne transmission competitiveness experiment, during which one variant may outperform the second, if the environmental condition favors sentinel susceptibility for the first over the second or if susceptibility of the sentinel changes between environmental conditions^42^. Also, a detailed understanding of the disease in the donor hamsters would have better elucidated the drivers of SARS-CoV-2 dynamics across environmental conditions.

There is still ongoing debate about the potential seasonality of SARS-CoV-2 in the human population. Its clarity might be confounded by the dominance of other epidemiological factors, such as population behavior, immunity, and ongoing virus evolution^22,23^. Our findings indicate that there is not a significant variation in the short-term aerosol transmission efficiency of SARS-CoV-2 at the various environmental conditions studied. This is corroborated by the lack of changes in the stability of SARS-CoV-2 aerosols at these conditions over 1 h. Even though we observed differences in the stability of SARS-CoV-2 at different environmental conditions, this was for a more extended exposure period of several hours to days^3–5^. This might indicate that environmental stability under these specific conditions will have a limited impact as a driver of SARS-CoV-2 seasonality.

## Material and methods

### Ethics statement

Approval of animal experiments was obtained from the Institutional Animal Care and Use Committee of the Rocky Mountain Laboratories. Performance of experiments was done following the guidelines and basic principles in the United States Public Health Service Policy on Humane Care and Use of Laboratory Animals and the Guide for the Care and Use of Laboratory Animals. Work with infectious SARS-CoV-2 strains under BSL3 conditions was approved by the Institutional Biosafety Committee (IBC). Inactivation and removal of samples from high containment was performed per IBC-approved standard operating procedures.

### Virus and cells

The SARS-CoV-2 variants used in this study are nCoV-WA1-2020 (MN985325, lineage A) and hCoV-19/USA/KY-CDC-2-4242084/2021 (EPI_ISL_1823618, Delta) obtained from CDC, Atlanta, USA. Virus propagation was performed in VeroE6 cells in Dulbecco’s Modified Eagle Medium (DMEM) supplemented with 2% fetal bovine serum (FBS), 2 mM L-glutamine, 100 U/mL penicillin and 100 μg/mL streptomycin (DMEM2). Cells were cultured in DMEM supplemented with 10% FBS, 2 mM L-glutamine, 100 U/mL penicillin, and 100 μg/mL streptomycin (DMEM10). No mycoplasma or contaminants were detected. All virus stocks were sequenced; and no SNPs compared to the patient sample sequence were detected.

### Environmental impact assessment

We assessed the possible physiological impact of the different environmental conditions on Syrian hamsters. Three sets of five four to 6-week-old Syrian hamsters (ENVIGO) were placed in transmission cages and the cages placed in the environmental chamber (to maintain and control temperature and relative humidity, (Caron Products)) at the following environmental conditions:

i. 10 °C, 45% relative humidity (RH) (temperate fall conditions)
ii. 27 °C, 65%RH (tropical conditions)
iii. 22 °C, 45%RH (climate-controlled indoor conditions).

For temperate fall and tropical conditions, the temperature/RH was dropped/increased incrementally for the first 3 days. Once the desired environmental condition was reached, the animals were left at the test condition for 6 additional days during which the activity of the hamsters was scored, and weights measured. The temperature of the animals was measured using BMDS IPTT-300 (Avidity Science) implantable transponders (implanted at the beginning of the experiment) and BMDS IPTT wand. Daily food and water intake were measured by determining the weight difference of food and water from those at day 0.

### Particle sizing

Particle sizing and transmission set-up are described in ref. ^30^. In summary, transmission cages were modified by introducing an inlet on the side wall of the infected hamster side, and sample ports on each end of the connection tube for measurement of particles in the air under constant airflow conditions. Particles were generated by spraying a 20% (v/v) glycerol solution with a standard spray bottle through the donor cage inlet. The particle size was measured using a Model 3321 aerodynamic particle sizer spectrometer (TSI). First, the donor cage was coated with three sprays at an interval of 30 s. The sample port was opened, and a sample was analyzed. Every 30 s a new spray followed, and five samples were analyzed (5 runs, each 60 s) for both donor side (primary infected side) and sentinel side.

### Virus inoculation

Before each transmission experiment, animals were acclimated to the test environmental condition for 7 days. The environmental condition (temperature and humidity) was adjusted each day sequentially to adapt the animal to the new environment in the first 3 days and allowed at the condition for 4 more days. After the transmission experiment, the process was reversed to bring animals back to room environmental conditions. After acclimatization to the environmental condition, Syrian hamsters were inoculated intranasally (I.N.) with 40 µL sterile DMEM containing either 8 × 10^4^ TCID_50_ SARS-CoV-2 nCoV-WA1-2020 (Lineage A) or hCoV-19/USA/KY-CDC-2-4242084/2021 (Delta variant). Hamsters were housed in groups of four animals and transmission was performed 1 day post inoculation (DPI).

### Transmission experiments

Transmission was conducted as described in ref. ^30^. Briefly, the aerosol transmission system consisted of two 7 in. × 1 in. × 9 in. plastic hamster boxes (Lab Products, Inc.) connected with a 3 in. diameter tube of 90 cm. Airflow was generated with a vacuum pump (Vacuubrand) attached to the box housing the naïve animals and was controlled with a float-type meter/valve (King Industries, McMaster-Carr). To ensure proper airflow from the donor box to the naïve box, the top of the naïve box was sealed while the filter top of the donor box remained open.

#### Assessment of the time window for transmission to occur at controlled laboratory conditions

Four donor animals were inoculated as described above and sentinels were exposed at 1 DPI to infected hamsters for 15 min or 1 h at a 1:1 ratio (donors were hosed in the donor side of the cage, sentinels in the naïve side). After exposure sentinel hamsters were individually housed and oropharyngeal swabs were taken in 1 mL DMEM with 200 U/ml penicillin and 200 µg/ml streptomycin for four days.

#### Comparison of environmental conditions during 1 h of exposure

After pre-conditioning, donor hamsters were intranasally inoculated and at 1 DPI the transmission experiment was performed by exposing the donor hamster to sentinels (1:1 ratio, eight pairs per environmental condition) for 1 h. After exposure, donor hamsters were swabbed, euthanized, and lung tissue harvested while the sentinels were moved to individual housing and reacclimatized to normal ambient conditions over 3 days. The sentinels remained singly housed at normal ambient condition until euthanasia at day 14 DPE.

### Viral RNA detection

Swabs from hamsters were collected as described above. Then, 140 µL was utilized for RNA extraction using the QIAamp Viral RNA Kit (Qiagen) using QIAcube HT automated system (Qiagen) according to the manufacturer’s instructions with an elution volume of 150 µL. Viral sub-genomic (sg) RNA was detected by qRT-PCR^48^. Five μL RNA was tested with TaqMan™ Fast Virus One-Step Master Mix (Applied Biosystems) using QuantStudio 6 Flex Real-Time PCR System (Applied Biosystems) according to instructions of the manufacturer. Ten-fold dilutions of SARS-CoV-2 standards with known copy numbers were used to construct a standard curve and calculate copy numbers/mL.

### SARS-CoV-2 titration

Viable virus in lungs or swabs was determined as previously described^13^. Briefly, tissues were weighted, then homogenized in 1 mL of DMEM with 2% FBS, 200 U/mL penicillin, and 200 µg/mL streptomycin. Virus titrations were performed by end-point titration in VeroE6 cells, inoculated with tenfold serial dilutions of hamster swabs or tissue homogenates in 96-well plates. Cytopathic effect was scored at day 6. TCID_50_ was calculated by the method of Spearman-Karber and, if required, adjusted for tissue weight.

### Serology

Serum samples were inactivated with γ-irradiation (according to laboratory SOPs prior to removal from the high containment lab). Maxisorp plates (Nunc) were coated with 50 ng spike protein per well and incubated overnight at 4 °C. After blocking with casein in phosphate-buffered saline (PBS) (ThermoFisher) for 1 h at room temperature (RT), serially diluted twofold serum samples (duplicate, in casein) were incubated for 1 h at RT. Spike-specific antibodies were detected with goat anti-hamster IgG Fc (horseradish peroxidase (HRP)-conjugated, Abcam) for 1 h at RT and visualized with KPL TMB 2-component peroxidase substrate kit (SeraCare, 5120-0047). The reaction was stopped with KPL stop solution (Seracare) and read at 450 nm. Plates were washed 3× with PBS-T (0.1% Tween) in between steps. The threshold for positivity was calculated as the average plus 3× the standard deviation of negative control hamster sera.

### SARS-CoV-2 stability in aerosol—Goldberg drum exposure

Droplet nuclei size particles (<5 µm) were generated using a 3-jet Collison nebulizer (CH Technologies) containing 10^5.75^–10^6.5^ TCID_50_/mL in 10 mL of DMEM supplemented with 2% FBS. The instrument’s humidity was prepared by using the integrated system of the Biaera unit. The targeted ambient temperature was maintained by keeping the drum in a pre-conditioned environmental chamber (Caron Products) for the duration of the experiment. Aerosols were upheld in suspension with a rotation of 3 mph to overcome terminal settling velocity.

Three independent replicates were performed for each of the environmental condition in a 1-h run for each of the SARS-CoV-2 variant assessed in this study. For each independent run, samples were collected at 0 and 1 h post aerosol generation. Samples were collected by drawing air at 6 liters per minute (LPM) for 30 s onto a 47 mm gelatin filter (Sartorius). Filters were dissolved in 10 mL of DMEM containing 10% FBS at 37 °C. Samples were frozen at −80 °C until assessment. Infectious virus titers were determined by end-point titration on VeroE6 cells.

### Statistical analysis

Significance tests were performed as indicated where appropriate: Kruskal-Wallis test followed by Dunn’s multiple comparisons test, Wilcoxon test, ordinary two-way ANOVA, followed by Šídák’s multiple comparisons test. Statistical significance levels are shown in the graph.

## Supplementary information

Supplementary Table 1

## Data Availability

The datasets generated and/or analyzed during the current study are available in the FigShare repository (10.6084/m9.figshare.24852228).
