# Supplementary material for: Airborne transmission efficiency of SARS-CoV-2 in Syrian hamsters is not influenced by environmental conditions
Source: Npj Viruses. 2024 Jan 9;2:2. doi: 10.1038/s44298-023-00011-3 (PMC11702665; doi:10.1038/s44298-023-00011-3)
Supplement: Supplementary file 1 — Supplementary Table 1 [file 44298_2023_11_MOESM1_ESM.docx]

**Airborne transmission efficiency of SARS-CoV-2 in Syrian hamsters is not influenced by environmental conditions**

Claude Kwe Yinda*, Julia R. Port*, Trenton Bushmaker, Jonathan E. Schulz, Shane Gallogly, Robert J. Fischer, Vincent J. Munster#

Laboratory of Virology, Division of Intramural Research, National Institute of Allergy and Infectious Diseases, National Institutes of Health, Hamilton, MT, USA

* These authors contributed equally

# Corresponding author. vincent.munster@nih.gov

Supplementary Table 1: Efficiency of airborne transmission of lineage A and Delta SARS-CoV-2 at different environmental conditions. Table show sgRNA at 1, 2, 3 day post exposure (DPE) and ELISA titers at 14 DPE. BLD = Below detection limit.

| Lineage A | | | | | | | | | | | | | | | | | | | | | | | | | |
| --- | --- | --- | --- | --- | --- | --- | --- | --- | --- | --- | --- | --- | --- | --- | --- | --- | --- | --- | --- | --- | --- | --- | --- | --- | --- |
|  | Animal ID | 1 | 2 | 3 | 4 | 5 | 6 | 7 | 8 | 1 | 2 | 3 | 4 | 5 | 6 | 7 | 8 | 1 | 2 | 3 | 4 | 5 | 6 | 7 | NA |
|  | Day | 27°C, 65%RH | | | | | | | | 22°C, 45%RH | | | | | | | | 10°C, 45%RH | | | | | | |  |
| sgRNA copies/mL (Log10) | 1 DPE | BLD | 4.959 | BLD | BLD | 6.17 | BLD | 5.548 | BLD | BLD | BLD | BLD | BLD | BLD | BLD | BLD | BLD | BLD | BLD | BLD | BLD | 3.556 | BLD | BLD |  |
|  | 2 DPE | 6.118 | 6.443 | BLD | BLD | 5.946 | BLD | 7.088 | BLD | BLD | BLD | BLD | BLD | 6.083 | BLD | 5.894 | 5.894 | BLD | 5.402 | BLD | BLD | 4.972 | BLD | 4.783 |  |
|  | 3 DPE | 6.163 | 6.656 | BLD | BLD | 6.303 | BLD | 6.331 | BLD | BLD | BLD | BLD | BLD | 6.132 | BLD | 6.291 | 6.279 | BLD | 5.922 | BLD | BLD | 5.809 | 5.303 | 5.007 |  |
| ELISA titer | 14 DPE | 76800 | 102400 | 0 | 0 | 102400 | 0 | 102400 | 0 | 0 | 0 | 0 | 0 | 102400 | 0 | 102400 | 76800 | 0 | 102400 | 0 | 0 | 51200 | 102400 | 102400 |  |
| Delta | | | | | | | | | | | | | | | | | | | | | | | | | |
|  | Animal ID | 1 | 2 | 3 | 4 | 5 | 6 | 7 | 8 | 1 | 2 | 3 | 4 | 5 | 6 | 7 | 8 | 1 | 2 | 3 | 4 | 5 | 6 | 7 | 8 |
|  | Day | 27°C, 65%RH | | | | | | | | 22°C, 45%RH | | | | | | | | 10°C, 45%RH | | | | | | | |
| sgRNA copies/mL (Log10) | 1 DPE | 5.877 | BLD | BLD | BLD | BLD | BLD | BLD | BLD | BLD | BLD | BLD | BLD | BLD | BLD | BLD | BLD | BLD | BLD | BLD | BLD | 4.078 | BLD | BLD | 4.505 |
|  | 2 DPE | 6.717 | BLD | BLD | BLD | BLD | BLD | BLD | 6.707 | 6.9 | 7.184 | 5.639 | BLD | BLD | BLD | 5.924 | BLD | BLD | BLD | BLD | BLD | 6.086 | 5.956 | BLD | 6.351 |
|  | 3 DPE | 7.277 | BLD | BLD | BLD | BLD | BLD | BLD | 6.479 | 6.876 | 6.413 | 6.323 | BLD | BLD | BLD | 5.392 | BLD | BLD | BLD | BLD | BLD | 4.744 | 6.377 | BLD | 6.215 |
| ELISA titer | 14 DPE | 25600 | 0 | 0 | 25600 | 0 | 0 | 0 | 51200 | 51200 | 51200 | 51200 | 51200 | 0 | 0 | 51200 | 0 | 0 | 0 | 0 | 6400 | 102400 | 25600 | 25600 | 102400 |
